# Host Bioenergetic Parameters Reveal Cytotoxicity of Antituberculosis Drugs Undetected Using Conventional Viability Assays

**DOI:** 10.1128/AAC.00932-21

**Published:** 2021-09-17

**Authors:** Bridgette M. Cumming, Zainab Baig, Kelvin W. Addicott, Dongquan Chen, Adrie J. C. Steyn

**Affiliations:** a Africa Health Research Institute, University of KwaZulu-Natal, Durban, KwaZulu-Natal, South Africa; b Division of Preventive Medicine and Comprehensive Cancer Center, University of Alabama at Birmingham, Birmingham, Alabama, USA; c Department of Microbiology, University of Alabama at Birmingham, Birmingham, Alabama, USA; d Centers for AIDS Research and for Free Radical Biology, University of Alabama at Birmingham, Birmingham, Alabama, USA

**Keywords:** bioenergetics, cytotoxicity, macrophages, tuberculosis drugs

## Abstract

High attrition rates in tuberculosis (TB) drug development have been largely attributed to safety, which is likely due to the use of endpoint assays measuring cell viability to detect drug cytotoxicity. In drug development for cancer, metabolic, and neurological disorders and for antibiotics, cytotoxicity is increasingly being assessed using extracellular flux (XF) analysis, which measures cellular bioenergetic metabolism in real time. Here, we adopt the XF platform to investigate the cytotoxicity of drugs currently used in TB treatment on the bioenergetic metabolism of HepG2 cells, THP-1 macrophages, and human monocyte-derived macrophages (hMDMs). We found that the XF analysis reveals earlier drug-induced effects on the cells’ bioenergetic metabolism prior to cell death, measured by conventional viability assays. Furthermore, each cell type has a distinct response to drug treatment, suggesting that more than one cell type should be considered to examine cytotoxicity in TB drug development. Interestingly, chemically unrelated drugs with different modes of action on Mycobacterium tuberculosis have similar effects on the bioenergetic parameters of the cells, thus discouraging the prediction of potential cytotoxicity based on chemical structure and mode of action of new chemical entities. The clustering of the drug-induced effects on the hMDM bioenergetic parameters are reflected in the clustering of the effects of the drugs on cytokine production in hMDMs, demonstrating concurrence between the effects of the drugs on the metabolism and functioning of the macrophages. These findings can be used as a benchmark to establish XF analysis as a new tool to assay cytotoxicity in TB drug development.

## INTRODUCTION

Although curative treatment is available for tuberculosis (TB), it requires adherence to a prolonged duration of drug therapy and exposes patients to drug-induced toxicities. The standard treatment for drug-susceptible TB includes rifampicin, isoniazid, ethambutol, and pyrazinamide over a 2-month intensive phase followed by a continuation phase of rifampicin and isoniazid for 4 months. Toxicities of anti-TB drugs have been reported in up to 80% of TB patients (1); the most common is hepatic toxicity associated with isoniazid, rifampicin, and pyrazinamide (2) in 2% to 28% of TB patients (3). Peripheral neuropathy from isoniazid occurs in up to 40% of TB patients (4) and more frequently among TB-HIV coinfected patients (5, 6). Ethambutol has commonly been reported to cause ocular toxicity, demonstrated by either optic or retrobulbar neuritis that is either reversible or irreversible (7–9). However, medications used to treat multidrug-resistant TB (MDR-TB) have much worse side effects (10). The most common adverse effects of the aminoglycosides include ototoxicity, vestibular toxicity (11, 12), nephrotoxicity, and electrolyte abnormalities (13, 14). Central nervous system adverse effects have been described in adults treated with fluoroquinolones (15, 16), in particular, with cycloserine (17, 18). Furthermore, peripheral neuropathy, including toxic optic neuropathy, is well documented among patients on prolonged linezolid treatment (19–21). Gastrointestinal intolerance and reversible hypothyroidism are known adverse effects of ethionamide and prothionamide during long-term therapy (22–24). The plethora of adverse effects is likely because most of the anti-TB drugs were discovered in the 1950s and 1960s, with new drugs only being discovered in the last 10 years (25). The side effects of anti-TB drugs impact adherence to chemotherapy and often result in relapse, treatment suspension, or failure and development of drug resistance. Thus, the development of new drugs with less cytotoxicity, shorter regimens, and fewer side effects is needed.

Although methodologies used to assess drug cytotoxicity in the early stages of drug development have evolved over the last few years, documented “cytotoxicity” is highly dependent on the type of assay and the cell type used to assay drug toxicity. These inconsistencies urge us to ask what defines cytotoxicity, what should be measured, and how can it be measured? Most standard cytotoxicity assays used in anti-TB drug development are endpoint assays measuring the viability or the integrity of the membranes of the cells. Viability assays include different classes of colorimetric tetrazolium reduction, resazurin reduction, protease markers, and ATP detection (26). Endpoint assays that assess the integrity of the cell membrane include the lactate dehydrogenase release assay and trypan blue exclusion assays. However, these assays only measure how the drug affects one of the cell’s parameters, of which, not all accurately represent the onset of cytotoxicity. Furthermore, these endpoint assays do not allude to alterations in the health of the cell that could potentially impact its functions in the absence of death. Nonetheless, defining the health status of a cell proves to be challenging, in that there is no clear delineation as to what we measure and how we measure it.

High attrition rates in recent drug development have been ascribed to safety issues with organ toxicity (27, 28), which has subsequently been proven to be due to or has strong evidence suggesting links to mitochondrial impairment (29–34). This has led to the development of several *in vitro* assays to measure mitochondrial function. Measurement of the oxygen consumption rate (OCR) in real time gives an indirect measurement of mitochondrial respiration. The extracellular flux (XF) analyzer (Agilent) enables high-resolution, real-time multiwell-plate readings of OCR in addition to real-time measurements of changes in the extracellular proton concentration to provide extracellular acidification rate (ECAR), which is considered an indirect measure of glycolysis. The response of OCR and ECAR to the consecutive addition of known mitochondrial and electron transport chain (ETC) modulators or stressors is used to calculate bioenergetic parameters associated with oxidative phosphorylation (OXPHOS) and metabolism of the cells, namely, basal respiration, basal ECAR, ATP-linked OCR, compensatory ECAR, maximal respiration, spare respiratory capacity, proton leak, and nonmitochondrial respiration (35). In some cases, these parameters can reveal drug cytotoxicity that is not detected in measurements of oxygen consumption rate (OCR) or extracellular acidification rate (ECAR) alone (36). ATP-linked OCR, determined from the decrease in basal respiration after the addition of an inhibitor of ATP synthase (complex V), oligomycin, has also been used to identify drugs that induce mitochondrial toxicity by reducing or inhibiting the activity of ATP synthase (37). Uncoupled respiration, induced by ionophores, results in maximal respiration that has been used as an indicator of the integrity of the electron transport chain (ETC) after drug treatment (38, 39). Extracellular flux analysis has been used to assess the cytotoxicity of drugs (30, 40–42) in the treatment of depression (43) and in the cancer field (44) as well as anesthetics (45), antibiotics (39, 46), and drugs for metabolic disorders (38). However, extracellular flux analysis has not yet been investigated as a potential platform to identify cytotoxic insults induced by anti-TB drugs.

The cell lines most often used to test the cytotoxicity of anti-TB drugs and new TB drug leads include the human hepatocellular carcinoma cell line HepG2, used to assess the hepatotoxicity of the drugs (47, 48), the human alveolar epithelial cell type 2 carcinoma cell line A549 (49–51), as the lung epithelium is the first lung surface coming into contact with Mycobacterium tuberculosis (52), the THP-1 human monocytic cell line (53, 54), and Vero (African green monkey kidney epithelial carcinoma) cells (55). Here, we adopted extracellular flux analysis as a rapid real-time platform to investigate the cytotoxicity of nine anti-TB drugs, individually and in combinations, currently used to treat drug-susceptible and multidrug-resistant TB on three human cell types, the HepG2 hepatocyte cell line, phorbol myristate acetate-differentiated THP-1 monocytes, and human monocyte-derived macrophages (hMDMs). Eight bioenergetic parameters calculated from the extracellular flux assay were compared to the viability results obtained from a 3-(4,5-dimethylthiazol-2-yl)-2,5-diphenyltetrazolium bromide (MTT) tetrazolium reduction assay. This assay measures the ability of NAD(P)H-dependent oxidoreductase enzymes to reduce a tetrazolium salt to formazan and is considered a measure of metabolism (56). These modulations of the bioenergetic parameters were compared with the effects of the anti-TB drugs on the functions of the hMDMs by measuring the cytokine levels in the supernatants of the hMDMs following treatment with the anti-TB drugs.

(This article was submitted to an online preprint archive [57].)

## RESULTS

### Experimental design.

**FIG 1:**
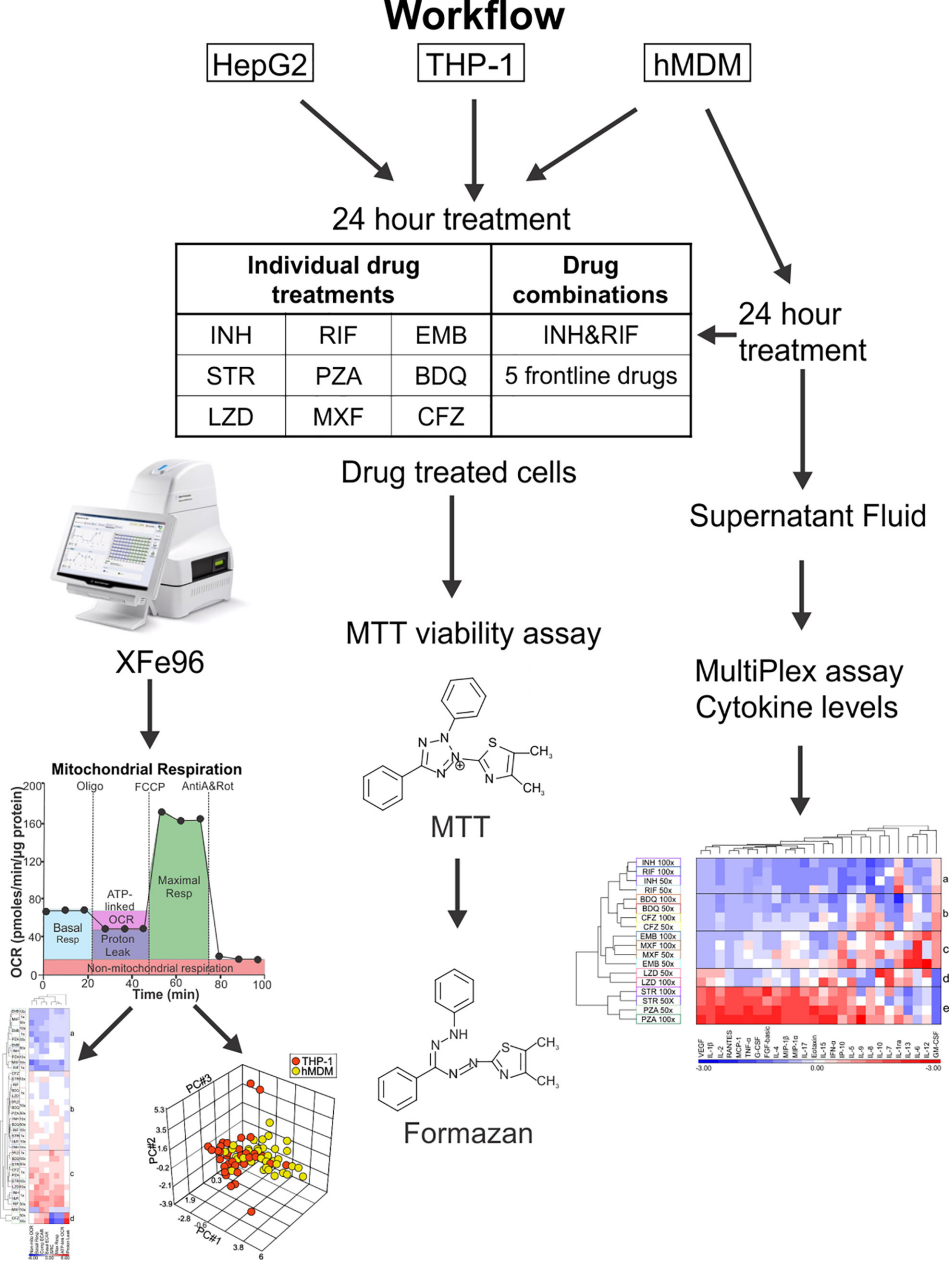
Workflow to assess extracellular flux analysis as a platform to assess the cytotoxicity of anti-TB drugs.

We explored the potential of extracellular flux analysis as a platform to assess the cytotoxic effects of anti-TB drugs on the energy metabolism of human cells according to the workflow diagram in Fig. 1. Three cell types (HepG2, THP-1 macrophages, and hMDMs) were treated with anti-TB drugs individually or in combination for 24 h. The effects of the anti-TB drugs on the cells were analyzed by (i) extracellular flux analysis to determine eight bioenergetic parameters, (ii) the MTT assay to determine viability, and (iii) cytokine analysis of the culture supernatant from the drug-treated hMDMs. As this study generated numerous parameters from three cell types treated with nine different drugs at four different concentrations, probability testing was not conducted. The resultant bioenergetic parameters, viabilities, and cytokine production of the drug-treated cells were analyzed using hierarchical clustering, Pearson’s correlation coefficient, and principal-component analysis (PCA).

The three cell types, HepG2 (hepatocytes), phorbol myristate acetate-differentiated THP-1 monocytes (THP-1), and human monocyte derived macrophages (hMDMs), were treated with nine anti-TB drugs individually or two drug combinations for 24 h (Fig. 1). HepG2 cells are a human hepatoma cell line that is commonly used to investigate altered hepatic metabolism and hepatic toxicity of new drug leads that are induced by mitochondrial dysfunction resulting from the drugs directly targeting the electron transport chain (58). Although primary human hepatocytes are considered the “gold standard” for assessing drug metabolism and toxicity (59), they have limited availability, short life spans, lose their hepatic phenotype in culture, and exhibit high variability in their genotype and drug-metabolizing enzymes (60–63). Despite the differences in glucose and lipid metabolism between the primary cells and the cell lines, the HepG2 cell line is still used for toxicology studies because it retains several liver functions (64, 65) and can be used for the detection of mitochondrial dysfunction (43, 66). Thus, we compared the effects of the anti-TB drugs on the bioenergetics of the HepG2 cells with those of two human macrophage cell types, human monocyte-derived macrophages (hMDMs) and a human THP-1 monocytic cell line that is differentiated with phorbol 12-myristate 13-acetate (PMA) to macrophages. We chose terminally differentiated human macrophages, as these are usually the first immune cells to come into contact with M. tuberculosis through the aerosolized route of infection. We did not use mouse macrophages because of the reported gene expression and metabolic differences between human and murine macrophages (67–70). Although metabolic differences have been observed between PMA-differentiated THP-1 cells and hMDMs (71), we have encountered large deviations in the bioenergetic responses of hMDMs derived from different donors. This donor-to-donor variation has been observed in other responses of hMDMs (72). Thus, responses of macrophages generated from the terminal differentiation of the human THP-1 monocytic cell line were also investigated.

The cells were treated with 1×, 10×, 50×, and 100× the MIC of the drug against M. tuberculosis, in the case of isoniazid (INH), rifampicin (RIF), pyrazinamide (PZA), ethambutol (EMB), moxifloxacin (MXF), clofazimine (CFZ) and linezolid (LZD), MIC_50_ in the case of bedaquiline fumarate (BDQ), and MIC_10_ in the case of streptomycin (STR). The MICs were used to enable comparisons among the effects of the different drugs on three cell types, as the physiological concentrations of the drugs vary between the serum and site of infection in addition to differing protein binding capacities of the drugs and variable drug absorption, metabolism, distribution, and perfusion of the infected areas among TB patients. As TB chemotherapy requires combination therapy to prevent the development of M. tuberculosis drug resistance and to combat the tendency of M. tuberculosis to persist in the face of drug treatment (73, 74), two sets of drug combinations were also examined at 1× and 10× MIC of the drugs in the combination. The first combination included all the drugs used in frontline treatment of drug-susceptible TB: INH, RIF, PZA, EMB, and STR (referred to here as 5FLD) (75). The second combination included just INH and RIF, as TB patients are treated with these two drugs for 4 months of their 6-month regimen.

### Bioenergetic parameters derived from extracellular flux analysis.

**FIG 2:**
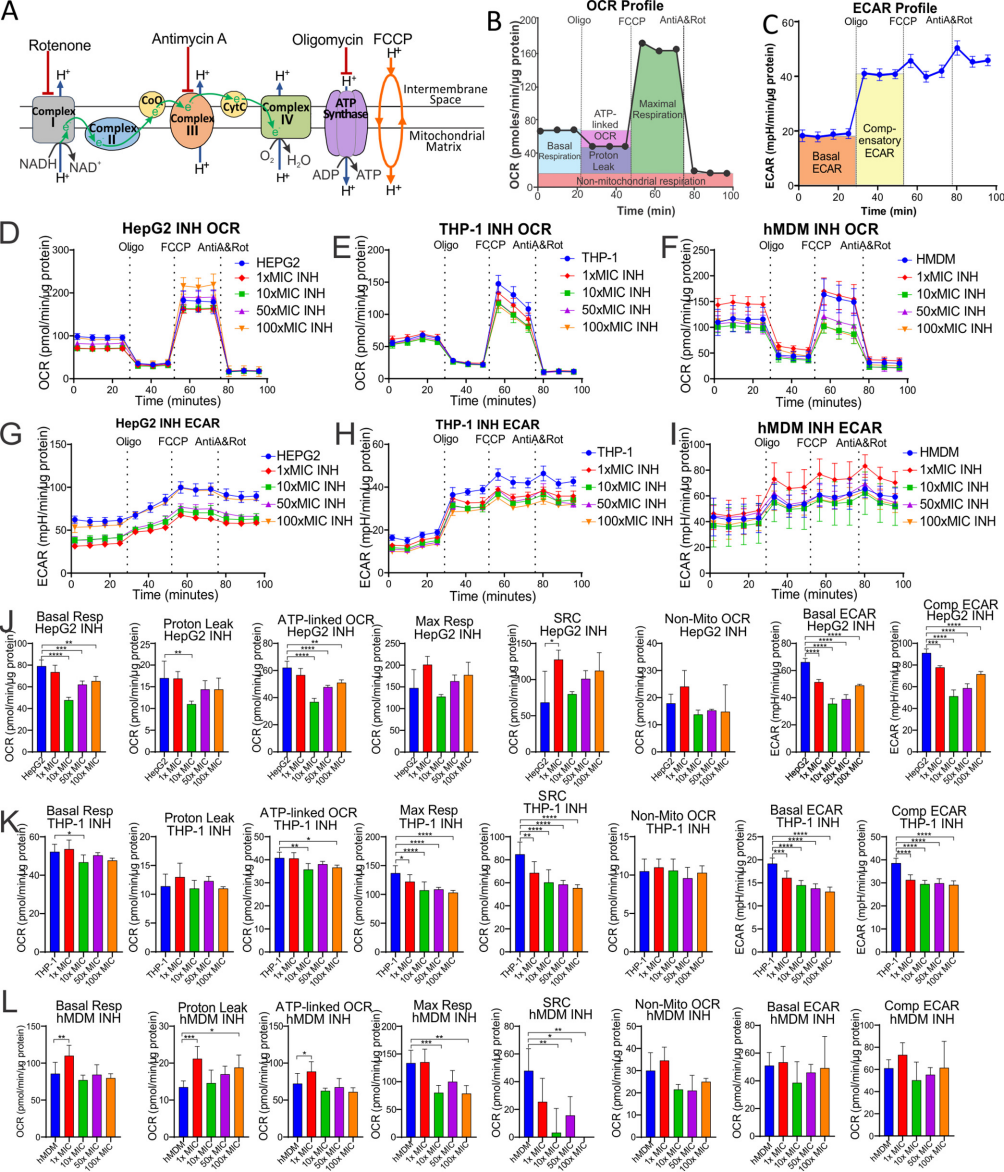
Bioenergetic parameters are calculated from the OCR and ECAR XF profiles of the CMST assay. (A) Modulators of the mitochondrial electron transport chain used to determine the bioenergetic parameters. (B) CMST profile demonstrating measurement of associated parameters of mitochondrial respiration. (C) ECAR profile demonstrating how basal ECAR and compensatory ECAR are measured from the CMST assay. Representative CMST profiles (D to F) and ECAR profiles (G to I) of HepG2 cells (D and G), THP-1 cells (E and H), and hMDMs (F and I) treated with 1×, 10×, 50×, and 100× MICs of INH for 24 h. Bioenergetic parameters of the HepG2 cells (J), THP-1 cells (K), and hMDM cells (L) treated with increasing MICs of INH for 24 h calculated from the representative profiles in panels D to I. Monocytes isolated from the buffy coats of three donors were mixed and used for each experiment (*n* = 1).

In the extracellular flux analysis, we used the Cell Mito stress test (CMST) on the XFe96 to calculate eight bioenergetic parameters of the untreated and drug-treated cells (Fig. 1). During the CMST run on the XFe96, mitochondrial modulators are added to the cells, and the resulting changes in oxygen consumption rate (OCR) and extracellular acidification rate (ECAR) are used to calculate the following bioenergetic parameters: the basal respiration (basal resp), ATP-linked OCR, proton leak, maximal respiration (max resp), spare respiratory capacity (SRC), nonmitochondrial respiration (non-mito OCR), basal extracellular acidification rate (basal ECAR), and compensatory extracellular acidification rate (comp ECAR) (Fig. 2A to C) (35).

Initially in the CMST, the respiration (OCR) of the untreated or drug-treated cells is measured to determine the basal respiration (Fig. 2B). This is followed by the addition of oligomycin, which inhibits complex V (ATP synthase) (Fig. 2A), to establish how much oxygen is consumed in the production of mitochondrial ATP by complex V. This ATP-linked OCR is equivalent to the decrease in the OCR following the addition of oligomycin (Fig. 2B). Subsequently, an ionophore, carbonyl cyanide 4-(trifluoromethoxy)phenylhydrazone (FCCP), is added to the cells, which allows protons to leak across the mitochondrial membrane into the matrix. This depolarizes the mitochondrial membrane potential, resulting in the ramping up of electron transport in the electron transport chain (ETC) to pump protons out of the matrix to reestablish the proton gradient and the mitochondrial membrane potential. The increased electron transport increases the oxygen consumption at complex IV and enables a measurement of the maximal respiration of the cell (Fig. 2A and B). After the addition of antimycin A and rotenone, inhibitors of complex III and complex I, the ETC is shut down, resulting in inhibition of mitochondrial OCR, and the resultant OCR gives a measurement of the nonmitochondrial respiration (Fig. 2A and B). This nonmitochondrial OCR is then subtracted from the basal respiration and the maximal respiration to give their true mitochondrial values. The difference between the nonmitochondrial respiration and the ATP-linked OCR is equivalent to the proton leak, which is the measure of oxygen consumption at complex IV that is not linked to ATP production and involved in restoring the mitochondrial membrane potential that is depolarized by the natural leak of protons into the mitochondrial matrix. SRC, which gives a measurement of the cell’s capacity to respire under conditions of stress, is calculated by the difference between the maximal respiration and the basal respiration. Extracellular acidification is generated by lactic acid that is derived from pyruvate, the end product of glycolysis, and carbonic acid that is derived from carbon dioxide produced by the tricarboxylic acid (TCA) cycle (76). ECAR measurements made simultaneously during the CMST run are used to determine the basal ECAR (ECAR prior to the addition of oligomycin) and compensatory ECAR (after the addition of oligomycin). Compensatory ECAR is induced by the inhibition of mitochondrial ATP synthase with oligomycin, which results in increased glycolysis to generate ATP for the cell’s demands (Fig. 2C).

Representative CMST OCR and ECAR profiles of each cell type are illustrated in Fig. 2D to I together with the altered profiles of the cells after treatment with increasing concentrations of isoniazid (INH). The calculated bioenergetic parameters from the HepG2 profiles in Fig. 2D and G are shown in Fig. 2J, those from the THP-1 profiles in Fig. 2E and H are shown in Fig. 2K, and those from the hMDM profiles in Fig. 2F and I are shown in Fig. 2L. These bioenergetic parameters provide quantitative measurements of aspects of mitochondrial respiration, nonmitochondrial respiration, and extracellular acidification, which enable assessment of how a potential drug affects the bioenergetic pathways of the cell that provide ATP. For instance, increasing concentrations of INH decreased both the basal resp and the ATP-linked OCRs of the HepG2 cells (Fig. 2J) and, to a lesser extent, the THP-1 cells (Fig. 2K), which are both essential for promoting OXPHOS. In both macrophage models, THP-1 cells, and hMDMs, INH decreased the max resp and the SRC that both give a measure of the cell’s ability to respond to conditions of stress (Fig. 2K and L). In hMDMs, INH increased the proton leak, which results in incomplete coupling of oxygen consumption and ATP synthesis in OXPHOS. Furthermore, INH decreased the basal ECAR and compensatory ECAR of both the HepG2 cells and the THP-1 cells (Fig. 2J and K), suggesting additional suppression of glycolysis. These findings suggest that INH depresses the bioenergetic pathways, particularly in the HepG2 cells and THP-1 cells.

However, moxifloxacin, for example, altered the bioenergetic parameters of these cell types differently (see Fig. S1 in the supplemental material). Although increasing concentrations of moxifloxacin (MXF) reduced the basal resp, max resp, and ATP-linked OCRs of the HepG2 cells and the hMDMs, it had no effect on these parameters in the THP-1 cells (Fig. S1C to E). Furthermore, MXF significantly reduced the basal ECAR and comp ECAR in all three cell types. The bioenergetic parameters of all three cell types in response to the anti-TB drug treatments are listed in Data Set S1.

In summary, we have adopted the extracellular flux analysis platform to assess the cytotoxic effects of anti-TB drugs on the bioenergetic metabolism of HepG2, THP-1, and hMDM cells. We have used the CMST assay, which generates eight bioenergetic parameters that reveal how anti-TB drugs modulate different aspects of respiration and glycolysis. Here, we have demonstrated how increasing concentrations of two of the drugs investigated, INH and MXF, altered the OCR and ECAR profiles of the three cell types and the resulting bioenergetic parameters. The responses of the bioenergetic parameters of each cell type to the drug treatment were analyzed using hierarchical clustering, Pearson’s correlation coefficient, and principal-component analysis (PCA) to identify any distinct trends.

### Each cell type has a unique bioenergetic fingerprint in response to the anti-TB drugs.

**FIG 3:**
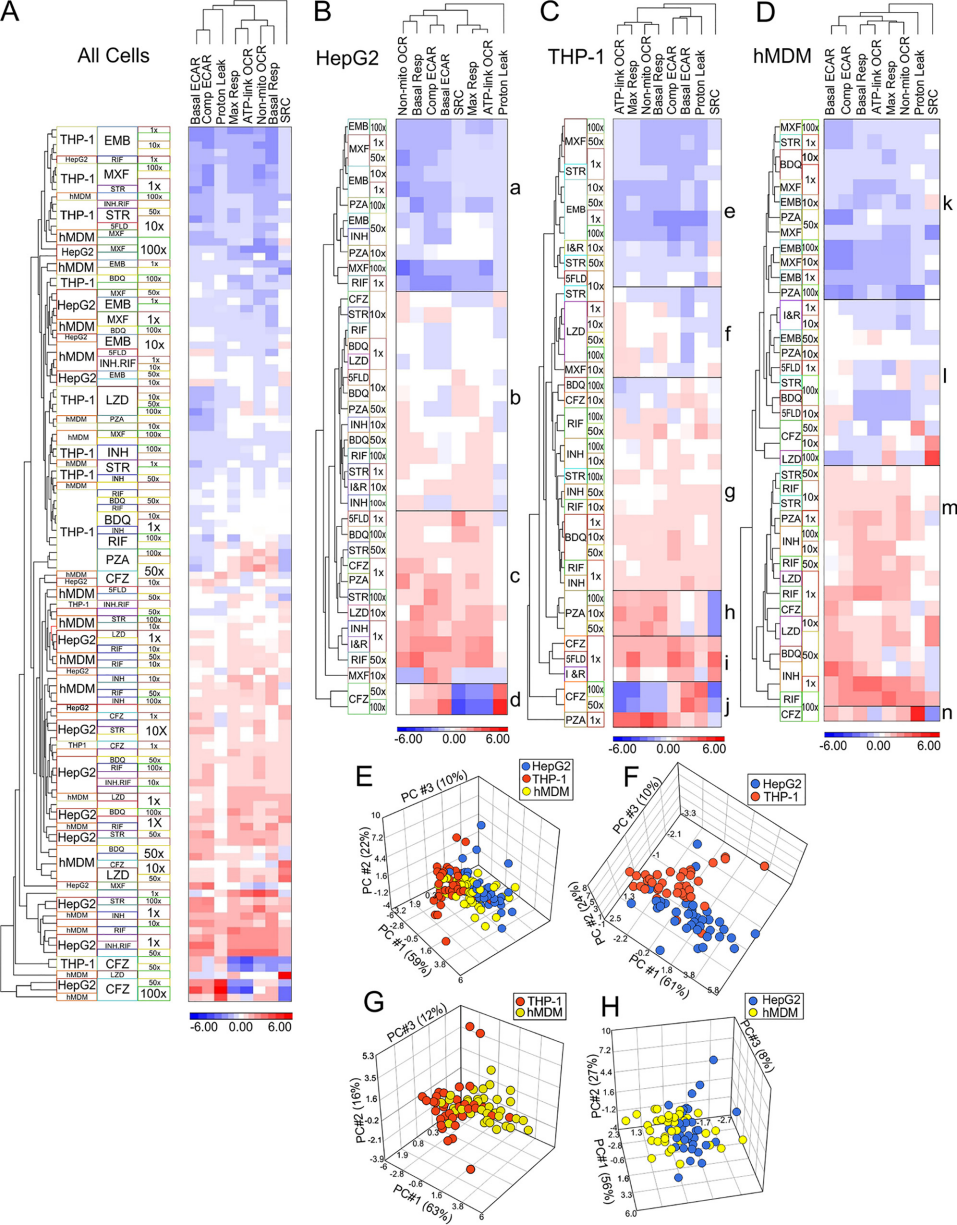
Each cell type demonstrates distinct bioenergetic fingerprints when treated with anti-TB drugs. (A to D) Heat maps of the hierarchical clustering of the z normalization values calculated from the relative bioenergetic parameters of all cell types (A), HepG2 cells (B), THP-1 cells (C), and hMDM cells (D) treated with the indicated concentrations of anti-TB drugs. PCAs of the combined bioenergetic parameters of all three cell types (HepG2, THP-1 and hMDM) (E), HepG2 and THP-1 cells (F), hMDM and THP-1 cells (G), and HMDM and HepG2 cells (H) treated with increasing MICs of anti-TB drugs.

Hierarchical clustering was used to determine if the bioenergetic metabolisms of the three cell types respond similarly to the anti-TB drugs. The three cell types were treated with four concentrations of nine anti-TB drugs individually and two concentrations of two drug combinations. After 24 h of treatment, their bioenergetic metabolism was examined using extracellular flux analysis and the CMST assay to calculate the eight bioenergetic parameters. A hierarchical cluster analysis of the bioenergetic parameters of the drug-treated cells relative to those of untreated cells was performed to assess if the drugs induced similar changes in the bioenergetic parameters of the different cell types. Both rows and columns of normalized relative values were used in the clustering, where Euclidean methods were applied for dissimilarities across both rows and columns. z normalization was performed to transform relative values of the bioenergetic values to an average of 0 and standard deviation (SD) of 1. Figure 3 shows heat maps of the z normalization values, where rows (drugs and their concentrations) and columns (bioenergetic parameters) have been ordered based on their correlation hierarchical clustering, using the average linkage method. Hierarchical clustering of the effects of the drugs on the bioenergetic parameters of all three cell types (Fig. 3A) demonstrates no clustering according to cell type or drug. This suggested that the measurement of eight bioenergetic parameters enabled the detection of a variety of effects on the bioenergetic processes in different cells.

The heat maps of the hierarchical clustering of the effects of the drugs on the bioenergetic parameters of the individual cell types (Fig. 3B to D) demonstrate that each cell type has distinctive patterns in their response to the drug treatments. These distinct patterns of clustering observed in each cell type have been indicated with boxes to facilitate discussion. In the HepG2 cells, the effects of all four concentrations of EMB, three of MXF, and two of PZA clustered together because they reduced the z normalization values of all the bioenergetic parameters to values lower the average (Fig. 3B, box a). The second cluster is divided into two groups with distinct patterns (Fig. 3B, boxes b and c). Both groups contain the same drugs but at different concentrations. The drugs include BDQ, INH, RIF, STR, LZD, PZA, and CFZ and the two drug combinations (5FLD and INH-RIF), but the concentrations of each drug are interspersed. This second cluster also includes the effects of 10× MIC of MXF, but they are separated from the effects of the other drugs in the second cluster. The effects of the 50× and 100× MICs of CFZ clustered separately from the effects of all the drugs (Fig. 3B, box d), with low z normalization values for maximal respiration, SRC, and ATP-linked OCR and high z normalization values for proton leak. In the HepG2 cells, proton leak was distinct from the clustering of the other bioenergetic parameters, with the ATP-linked OCR and the max resp being the most closely linked parameters.

In the THP-1 cells, there was a great deal more clustering of the effects of several concentrations of the same drug on the bioenergetic parameters than that detected in the HepG2 cells. This suggests that some of the anti-TB drugs have very distinct effects on the THP-1 cells that are not observed in the HepG2 cells. For example, the effects of all four concentrations of EMB and of LZD clustered distinctly together in Fig. 3C, boxes e and f, respectively. The effects induced by three concentrations each of MXF, BDQ, and PZA clustered together distinctly (Fig. 3C, boxes e, f, and g, respectively), and two concentrations each of INH, RIF, and CFZ clustered together. However, THP-1 cells behaved similarly to the HepG2 cells in that the effects of EMB and MXF clustered together because they reduced the z normalization values below normal (Fig. 3C, box e). Yet, in the case of the THP-1 cells, the effects of MXF and EMB clustered together with those of two STR concentrations and the 10× MICs of both drug combinations, 5FLD and INH-RIF (Fig. 3C, box e). The “block” showing the effects of the four concentrations of LZD clustered with the effects of the 10× MICs of both STR and MXF (Fig. 3C, box f). This cluster (box f) was closely linked to the effects of BDQ, RIF, and INH, which clustered together with 100× MIC STR and 10× MIC CFZ (Fig. 3C, cluster g) by inducing small or no fluctuations in z normalization values from the average. Strikingly, the effects of three PZA concentrations clustered on their own, inducing higher than average z normalization values in the ATP-linked OCR, max resp, non-mito OCR, and basal resp (Fig. 3C, box h). The 1× MIC of PZA had the most divergent effects on the bioenergetic parameters, such that it clustered separately from all the other drugs (Fig. 3C, last row in box j). The effects of the lowest concentrations of CFZ and of both drug combinations, 5FLD and INH-RIF, clustered together (Fig. 3C, box i), with the effects of the 50× and 100× MICs of CFZ also clustering apart from the effects of the other drugs (Fig. 3C, box j) by inducing lower than average z normalization values for the ATP-linked OCR, max resp, non-mito OCR, and basal resp. The hierarchical clustering of the bioenergetic parameters indicates that the effects of the drugs on the SRC of the THP-1 cells cluster separately from the other bioenergetic parameters.

Treatment of the hMDM cells with the anti-TB drugs appears to have induced lower z normalization values than those observed in the other two cell types (Fig. 3D). As in the case of the THP-1 cells, the effects of MXF and EMB clustered together with the effects of 1× MIC of STR, but in the hMDMs, they also clustered with the low concentrations of BDQ and higher concentrations of PZA, because they all reduced the z normalization values below average (Fig. 3D, box k). This cluster (box k) was closely linked to a cluster of the effects of the two drug combinations, 5FLD and INH-RIF and other concentrations of BDQ, PZA, and STR that had some minimal fluctuations in the z normalization values from the average (Fig. 3D, box l). The effects of 50× and 10× MICs of CFZ and 100× MIC LZD clustered separately but were still linked to previously described cluster (Fig. 3D, box l). In contrast, the effects of RIF, INH, LZD, and remaining concentrations of STR, PZA, and BDQ clustered together by increasing the z normalization values of the bioenergetic parameters to values higher than average (Fig. 3D, box m). Lastly, similarly to both the HepG2 and THP-1 cells, the effects of the 100× MIC CFZ clustered separately from the effects of all the other drugs (Fig. 3D, box n). Similar to that in the THP-1 cells, the drug-induced changes in the SRC of the hMDMs clustered separately from the other bioenergetic parameters.

The relatedness between the cell types was examined using PCA (77) of the effects of the four concentrations of the anti-TB drugs on the combined relative bioenergetic parameters of each cell type (Fig. 3E). When the PCAs of the cell types were compared pairwise, there was a noticeable separation between the effects of the drugs on the bioenergetic parameters of the HepG2 and THP-1 cells (Fig. 3F) that was not as well defined when the hMDM cells were compared with the THP-1 cells (Fig. 3G) or with the HepG2 cells (Fig. 3H). This illustrates that the anti-TB drugs do not have equal effects on all cell types, and other cell types should be considered when assessing cytotoxicity/modulatory effects of anti-TB drugs.

In sum, the hierarchical clustering and PCA of the bioenergetic parameters generated from the extracellular flux analysis revealed that the three cell types investigated demonstrate unique bioenergetic responses to treatments with the anti-TB drugs. Hierarchical clustering demonstrated different patterns of clustering of the bioenergetic parameters of the three cell types when treated with the anti-TB drugs. In particular, PCA revealed that the bioenergetic parameters of the drug-treated THP-1 cells had more noticeable separation from the HepG2 cells than from the hMDM cells.

### Basal respiration correlates with all other bioenergetic parameters in all three cell types.

**FIG 4:**
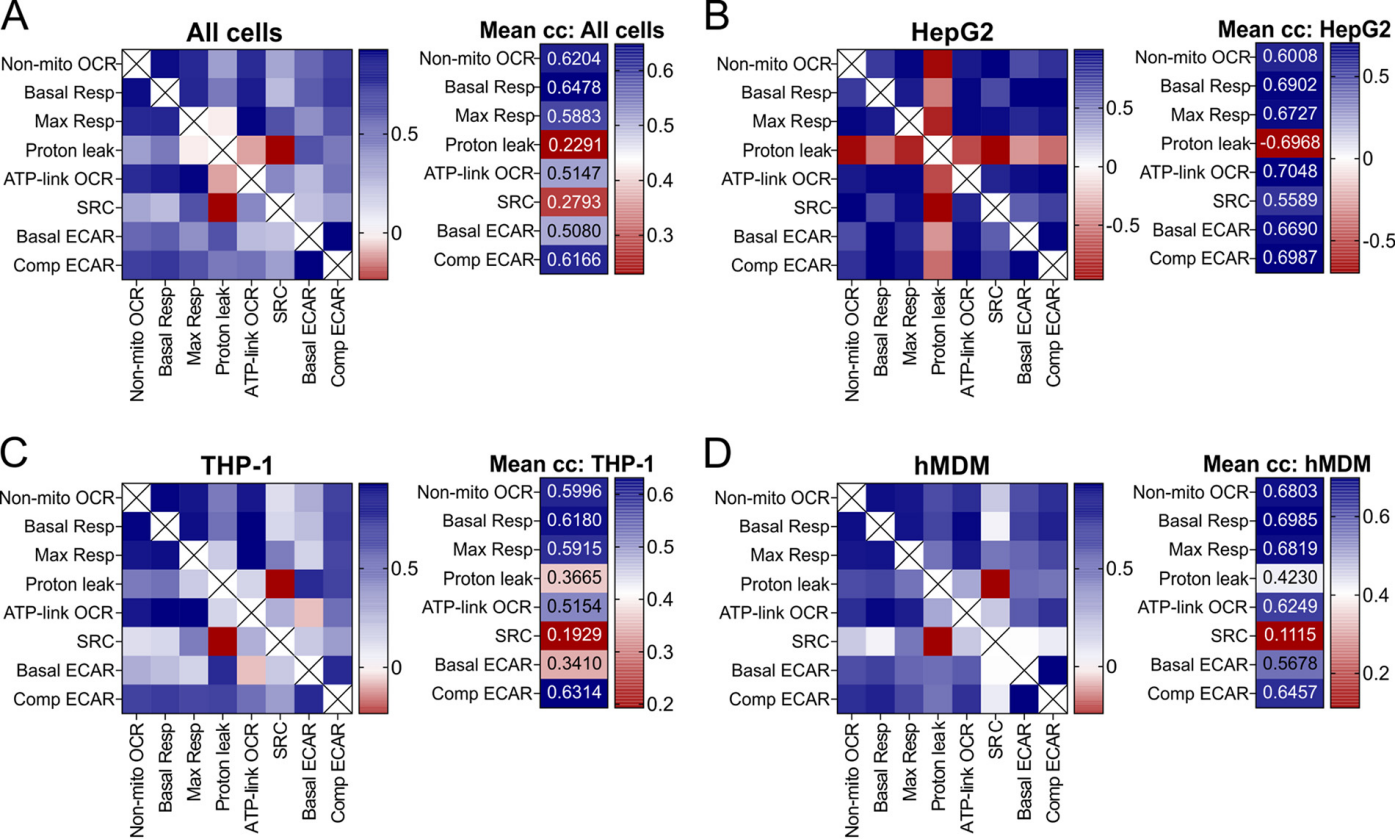
Basal respiration correlates with all the bioenergetic parameters. Heat maps of the Pearson correlation coefficients of the averaged bioenergetic parameters induced by anti-TB drug treatment (left) and the means of the correlation coefficients (right) of all cell types (A), HepG2 cells (B), THP-1 cells (C), and hMDMs (D) treated with increasing MICs of anti-TB drugs. (Refer to Data Sets S2 to S5 in the supplemental material for the Pearson correlation coefficients used to plot the heat maps.)

To identify a bioenergetic parameter that correlates with the changes in the other bioenergetic parameters in response to treatment with any of the anti-TB drugs, Pearson’s correlation coefficients were calculated for all pairwise combinations of the combined bioenergetic parameters of all three cell types combined (Fig. 4A) and for each cell type individually (Fig. 4B to D). The left panel in Fig. 4A shows a heat map of the correlation coefficients of each pairwise comparison of the combined bioenergetic parameters for all the cells, and in the right panel, the averages of these correlation coefficients for each bioenergetic parameter are given in a heat map to demonstrate the parameters with the highest correlations. The Pearson’s correlation coefficients for the bioenergetic parameters of each cell type were demonstrated in a similar manner (Fig. 4B to D).

When the bioenergetic parameters of all cell types were analyzed, the relative basal resp had the highest correlation coefficient (*r* = 0.64) (Fig. 4A) with all the other bioenergetic parameters of all three cell types. In the case of the HepG2 cells, several bioenergetic parameters behaved similarly to each other in response to the anti-TB drugs, with relative ATP-linked OCR and relative comp ECAR having the highest correlation coefficient (*r* = 0.70) (Fig. 4B), followed by relative basal resp (*r* = 0.69). Notably, the proton leak in the HepG2 cells exhibited a strong negative correlation with all the other bioenergetic parameters. This demonstrates strong inverse relationships between proton leak and the other bioenergetic parameters of the HepG2 cells when treated with the anti-TB drugs. In THP-1 cells, the relative comp ECAR (*r* = 0.63) (Fig. 4C) and basal resp (*r* = 0.61) had the highest correlations with the other bioenergetic parameters in response to the anti-TB drugs. In hMDM cells, relative basal resp correlated with the changes in the other respiratory parameters in response to the anti-TB drugs (*r* = 0.70) (Fig. 4D), with max resp and non-mito OCR also having a high correlation (0.68 in both cases).

Conversely, the bioenergetic parameters with the lowest correlations with the other bioenergetic parameters were proton leak in HepG2 cells (*r* = −0.70) (Fig. 4B), SRC in THP-1 cells (*r* = 0.20) (Fig. 4C), and SRC in hMDMs (*r* = 0.11) (Fig. 4D). When the bioenergetic parameters of all the cells were combined, proton leak had the lowest correlation (*r* = 0.23) (Fig. 4A), followed by the SRC (*r* = 0.30). This suggests that SRC and proton leak respond differently than the other parameters to anti-TB drug treatment, possibly due to increased sensitivity. This is feasible with SRC, given that SRC is a measure of the ability of the cell to respire under conditions of stress, which may be compromised with the anti-TB drug treatment.

Overall, we conclude that basal resp can be used to assess the overall changes in the bioenergetic metabolism induced by the anti-TB drugs, as it has a high correlation coefficient with the effects of the anti-TB drugs on the other bioenergetic parameters among all three cell types and has the highest correlation coefficient in hMDMs. Another parameter that demonstrated high correlation with the other bioenergetic parameters in all three cell types was comp ECAR, being the parameter with the highest correlation in THP-1 cells. In HepG2 cells, which are more oxidative than glycolytic, ATP-linked OCR is the parameter that had the highest correlation with the other bioenergetic parameters. SRC and proton leak demonstrated the least correlation with the other bioenergetic parameters, probably in response to different mechanisms of toxicity induced by the anti-TB drugs.

### The bioenergetic parameters enable separation of distinct effects of anti-TB drugs.

**FIG 5:**
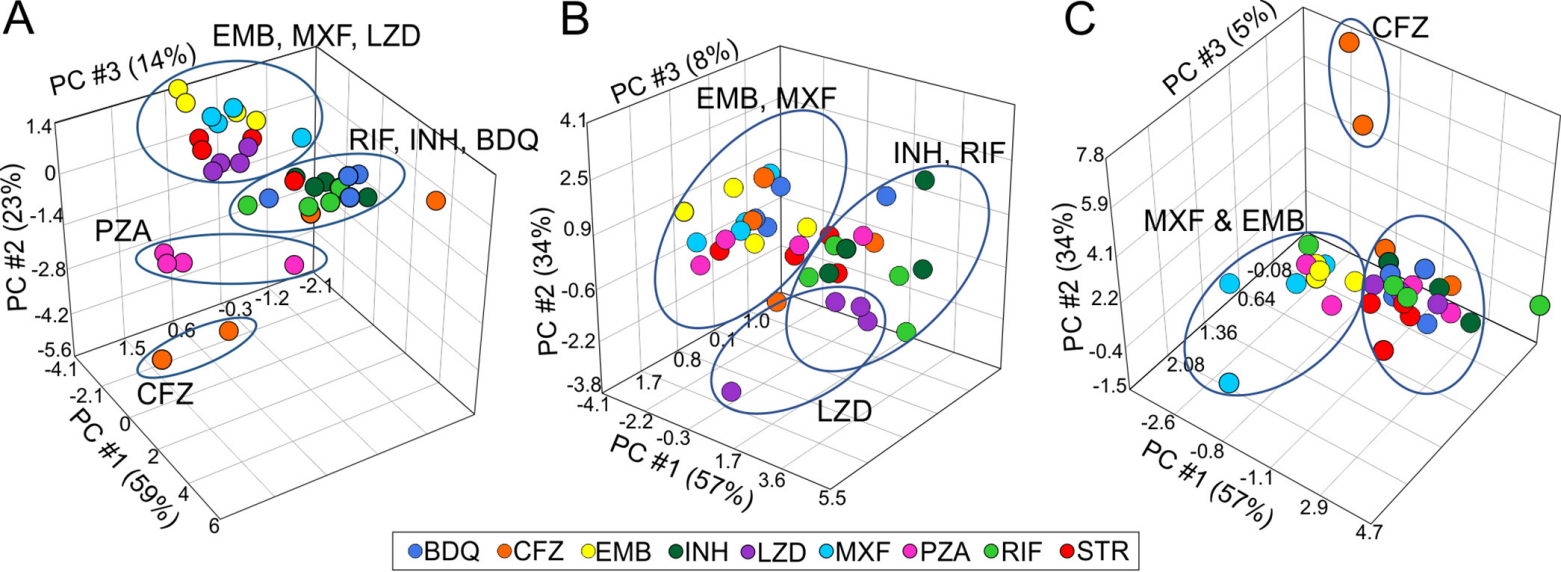
The bioenergetic parameters can be used to distinguish the effects of anti-TB drugs on the cells. PCAs of the averaged bioenergetic parameters of the THP-1 cells (A), HMDM cells (B), and HepG2 cells (C) that were treated with increasing concentrations of anti-TB drugs, demonstrating clustering of the different drug treatments. The color of the spheres indicates different drug treatments.

To identify if groups of anti-TB drugs have similar effects on the bioenergetic parameters of each cell type, the identity of the anti-TB drugs was selected in the PCA for each cell type. Clustering of the drug effects on the bioenergetic parameters of the cell was most distinct in the THP-1 cells. Figure 5A shows how the modulation of the THP-1 bioenergetic parameters induced by EMB, MXF, and LZD clustered separately from the effects of RIF, INH, and BDQ on the THP-1 cells. The effects induced by PZA on the bioenergetics of the THP-1 cells are separate from the EMB-MXF-LZD and RIF-INH-BDQ clusters. The effects of the highest concentrations of CFZ (50× and 100× MICs) on THP-1 bioenergetics are even further removed from the effects of the other drugs. This reflects the hierarchical clustering of the effects of the drugs on the THP-1 bioenergetic parameters observed in the heat map in Fig. 3C.

In hMDM cells, the effects of EMB and MXF on the bioenergetics are again separated from the effects of INH and RIF on the hMDM bioenergetics (Fig. 5B). Specific to the hMDMs, the effects of LZD on the bioenergetics separated from the bulk of the effects of the other TB drugs. The effects of 100× MIC of CFZ were detached from the bioenergetic modulations generated by the other anti-TB drugs. This is supported by the hierarchical clustering of the effects of the drugs on the hMDM bioenergetic parameters in Fig. 3D.

In the HepG2 cells, the separation of the effects on the anti-TB drugs on the bioenergetic parameters were less defined (Fig. 5C). As in the THP-1 and hMDM cells, the bioenergetic parameters of the MXF- and EMB-treated HepG2 cells were separated from the bioenergetic parameters of most of the other drug-treated cells (Fig. 5C). Again, the bioenergetic modulations induced by the highest concentrations of CFZ were clearly disconnected from the effects of the other anti-TB drugs on the bioenergetics of the HepG2 cells. The clustering of MXF and EMB and the disconnection of CFZ align with the hierarchical clustering of the effects of the anti-TB drugs on HepG2 cells. Overall, these findings indicate that the bioenergetic parameters can be used to identify groups of drugs with similar effects on the bioenergetic metabolism of the cells.

### The bioenergetic parameters reveal a broader range of effects than percent viability.

**FIG 6:**
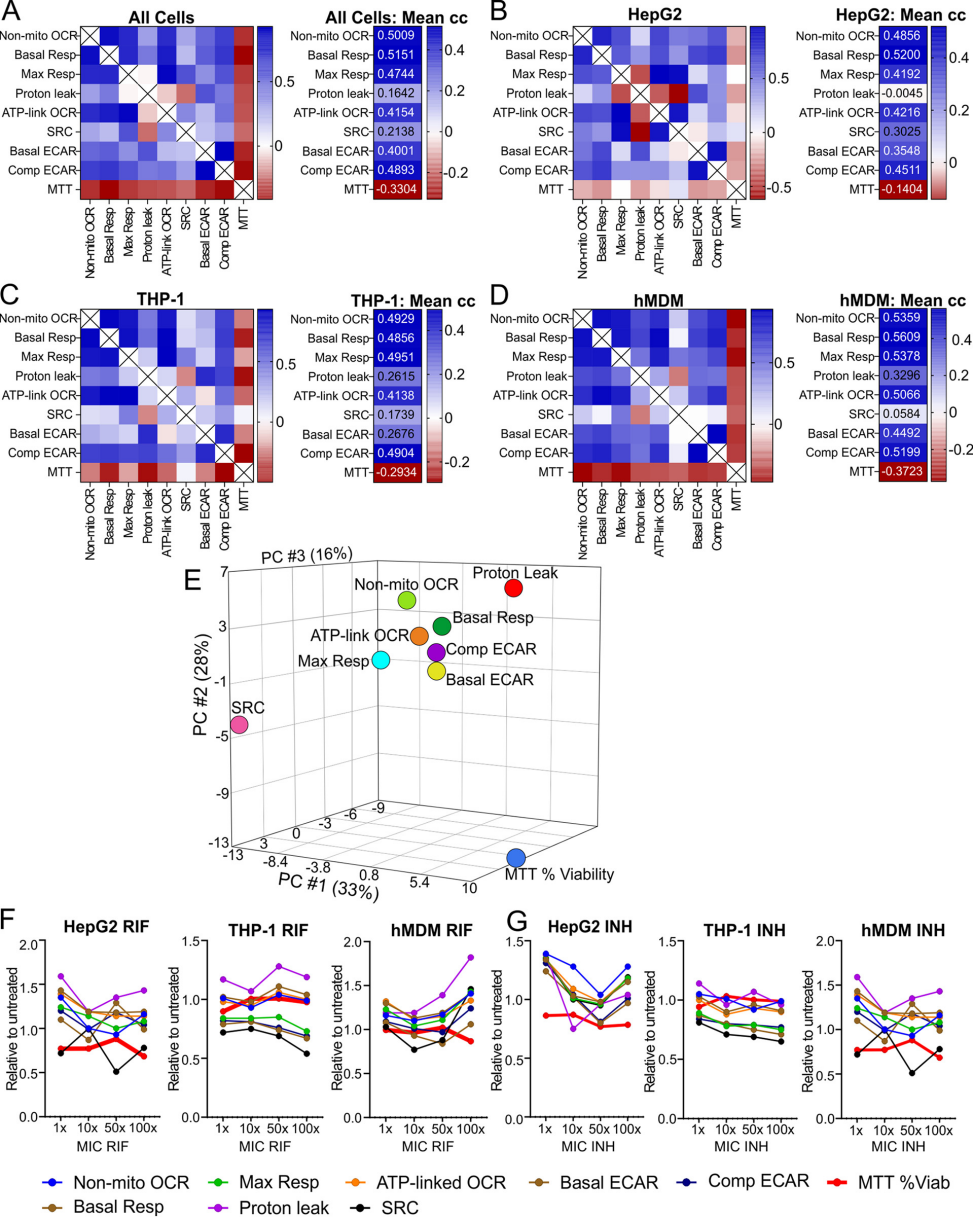
The bioenergetic parameters display a wider range of effects of the anti-TB drugs than the MTT assay. (A to D) Heat maps of the Pearson’s correlation coefficients of MTT percent viability correlated with the averaged bioenergetic parameters pairwise (left) and an adjacent heatmap of the means of the correlation coefficients (right) of all cell types (A), HepG2 cells (B), THP-1 cells (C), and hMDMs (D) treated with increasing MICs of anti-TB drugs. (Refer to Data Sets S6 to S9 for the values of the Pearson correlation coefficients used to plot the heat maps.) (E) PCA depicts separation of the cumulative MTT percent viability values from the averaged individual bioenergetic parameters. Line plots of relative bioenergetic parameters and the MTT percent viability of the three cell types treated with increasing MICs of RIF (F) and INH (G).

As viability assays have conventionally been used to assess the cytotoxicity of new drug leads, we compared the percentage viabilities obtained in MTT assays (Supplemental Data Set S10) for each drug-treated cell type with the bioenergetic parameters obtained under the same conditions. We calculated the Pearson’s correlation coefficients for all possible pairwise combinations of the bioenergetic parameters with the percent viabilities. Heat maps in Fig. 6A to D reveal that viabilities of all the drug treatments negatively correlated with all the averaged bioenergetic parameters when the parameters of all three cell types were combined and separated by cell type. These negative correlations were all less than −0.4.

This poor correlation between the percent viability and the bioenergetic parameters of the cells was supported by PCA of the bioenergetic parameters and percent viabilities of all the cell types combined. Figure 6E demonstrates the clear separation of the percent viabilities of the MTT assay from all the bioenergetic parameters. Furthermore, PCA shows that the relative SRC is separated to the greatest degree from the other bioenergetic parameters, which are clustered to some extent. The SRC is separated to the greatest degree from proton leak. This is also supported by the Pearson correlation analysis, where the relative SRC had the lowest positive correlation coefficient with the other bioenergetic parameters in both the THP-1 and hMDM cells (Fig. 6C and D). However, in HepG2 cells and in the combination of the bioenergetic parameters of all three cell types, relative proton leak had the lowest positive correlation coefficient, followed by SRC (Fig. 6A and B).

Line plots of the individual relative bioenergetic parameters and percent viabilities versus concentrations of the drugs demonstrate variability in the parameters and percent viabilities with an increase in drug concentration (see Fig. S2). Figure 6F and G show representative line plots of the bioenergetic parameters and the percent viability (thick red lines) of the three cell types when treated with RIF (Fig. 6F) or with INH (Fig. 6G). The line plots demonstrate that the bioenergetic parameters of the cells exhibit a much broader range of effects than that observed in the percent viability with an increase in drug concentration.

In summary, the bioenergetic parameters reveal changes in the energy metabolism of the three cell types induced by the anti-TB drugs that are not reflected by changes in the percent viability measured using the MTT assay. This is due to the extracellular flux analyzer being an extremely sensitive instrument, in that it measures oxygen consumption in picomoles O_2_ per minute and extracellular acidification in mpH per minute, in addition to generating eight bioenergetic parameters from the addition of metabolic modulators. In comparison, the MTT assay measures the activity of one metabolic enzyme, NADPH-dependent cellular oxidoreductase, in the cells as a measure of the metabolic activity and viability. This suggests that the extracellular flux analysis is more sensitive than the MTT assay for assessing cytotoxic effects of drugs on the cell.

### Clustering of the effects of anti-TB drugs on hMDM cytokine production resembles that observed for the hMDM bioenergetic parameters.

**FIG 7:**
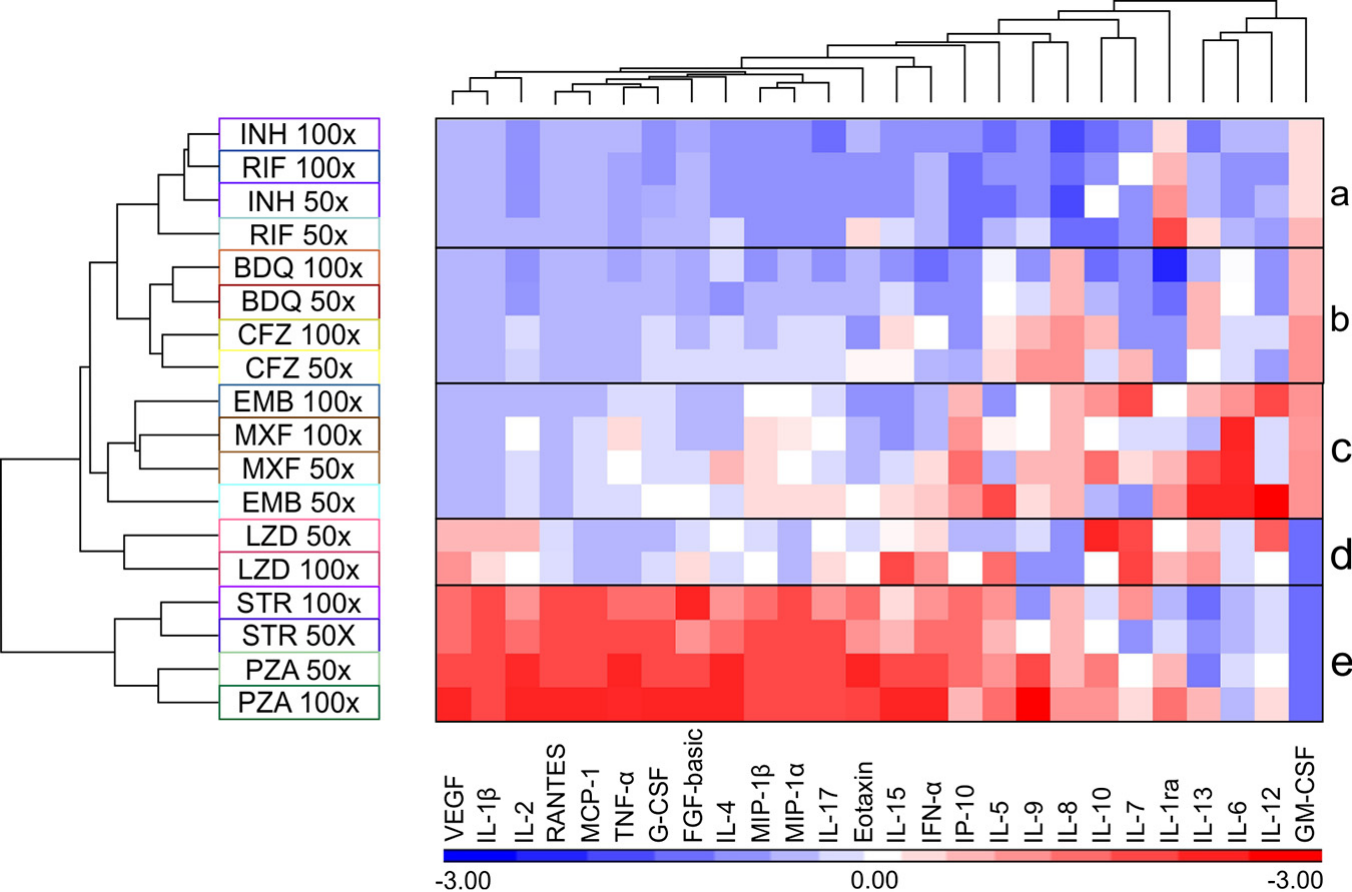
The anti-TB drugs alter cytokine production of the hMDM cells in a pattern resembling that of the hMDM bioenergetic parameters. Heat map and hierarchical clustering of the z normalization values of the cytokines produced by hMDMs after treatment with the indicated concentrations of the anti-TB drugs.

As metabolism has been demonstrated to be intricately related to immunity (78), we investigated the effects of the anti-TB drugs on the cytokine production by the hMDMs. To determine the effects of the anti-TB drugs on the function of the macrophages, cytokine production by hMDMs after treatment with the two highest concentrations of each drug was assessed in a multiplex assay. Hierarchical cluster analysis of the cytokine production by the drug-treated hMDMs relative to that by the untreated hMDMs was performed. Figure 7 shows a heat map of the z normalization values, where rows (drugs and their concentrations) and columns (cytokines) have been clustered based on their correlation hierarchical clustering or similarities, using the average linkage method.

As observed in the hierarchical clustering of the bioenergetic parameters (Fig. 3D) and the PCA (Fig. 5B) of the hMDMs treated with anti-TB drugs, the effects of INH and RIF on cytokine production clustered together, reducing the z normalization values for cytokine production below the average of that for all the drugs investigated (Fig. 7, box a). The only exceptions are the z normalization values of interleukin-1 receptor antagonist (IL-1Ra) and granulocyte-macrophage colony-stimulating factor (GM-CSF), which were increased above average in the case of IL-1Ra, with only slight increments in GM-CSF. IL-1Ra is the IL-1 receptor antagonist and it inhibits the proinflammatory action of IL-1 (79). Linked to the effects of INH and RIF are the effects of BDQ and CFZ that clustered together (Fig. 7, box b). Likewise, BDQ and CFZ also reduced the z normalization values of most of the cytokines to values below the average. Cytokines that were increased above the average include IL-8 and GM-CSF by both CFZ and BDQ, IL-9 by CFZ, and slight increases in IL-13. GM-CSF stimulates the differentiation and proliferation of myeloid progenitors in the bone marrow and induces the activation and migration of myeloid progenitors to sites of inflammation (80). IL-8 is a chemokine that attracts neutrophils to regions of inflammation and activates them. Both IL-9 and IL-13 are Th2 cytokines that inhibit the proinflammatory response (81, 82), but IL-9 promotes mast cell growth and function (83), and IL-13 mediates allergic inflammation and asthma (82).

The two clusters mentioned above were linked to the clustered effects of EMB and MXF on hMDM cytokine production. These drugs induced both a decrease and an increase in the z normalization values relative to the average, with a particularly high induction of the z normalization values of IL-6 and IL-13. EMB also increased the values for IL-12, a proinflammatory cytokine (Fig. 7, box c). The clustering of cytokine production resulting from treatment of the hMDM cells with EMB and MXF is supported by the hierarchical clustering of the effects of EMB and MXF on the bioenergetic parameters of the hMDM cells (Fig. 3D, box k) and the clustering observed in the PCA of the effects of MXF and EMB on the bioenergetic parameters of the hMDM cells (Fig. 5B). LZD clustered on its own, because it increased the z normalization values of different cytokines, in particular, IL-7 and IL-10, at 50× MIC, with slight increases in vascular endothelial growth factor (VEGF) and IL-1β (Fig. 7, box d). This also correlates with the distinct clustering of the effects of all four concentrations of LZD on the bioenergetic parameters of the hMDM cells in the PCA in Fig. 5B apart from the effects of the other drugs. In contrast to the previous drugs, it reduced the z normalization values of GM-CSF. Lastly, STR and PZA clustered together, with a striking inverse of the patterns of the z normalization values observed in the previous clusters (Fig. 7, box e). These cytokines with high z normalization values include proinflammatory cytokines such as IL-1β, IL-2, tumor necrosis factor alpha (TNF-α), gamma interferon (IFN-γ), and IL-17 and chemokines such as monocyte chemoattractant protein 1 (MCP-1), macrophage inflammatory protein 1 alpha (MIP-1α), MIP-1β, and interferon gamma-induced protein 10 (IP-10). This suggests that STR and PZA potentially induce proinflammatory modulation of the macrophages. However, like LZD, STR and PZA also reduced the z normalization values of GM-CSF.

In summary, the hierarchical clustering of the cytokines produced by the anti-TB drug-treated hMDM cells resembled the clustering observed in the bioenergetic parameters of the drug-treated hMDMs in the PCA, especially in the case of RIF, INH, EMB, MXF, and LZD. Interestingly, treatment of the hMDM cells with STR or PZA induced a pattern of cytokine production completely different from that with the other anti-TB drugs, suggesting induction of a proinflammatory response.

## DISCUSSION

High attrition rates in drug development during the clinical and postmarket phases due to safety issues underscore the need for new technology to screen for “cytotoxicity” at early stages in drug development. In anti-TB drug development, current methods used to assess cytotoxicity of new chemical entities have a single endpoint measurement as an indicator of cell viability, which does not reflect earlier events of distress induced by the compounds in the absence of cell death. Here, we adopted a multiwell noninvasive extracellular flux analysis platform that rapidly detects the modulation of bioenergetic metabolism of cells induced by anti-TB drugs in real time prior to cell death that is measured by conventional viability assays. This rapid detection of earlier events of anti-TB drug-induced bioenergetic distress provides a novel tool to detect early toxicity affecting the health of the cells that potentially leads to cellular dysfunction, thereby reducing high attrition and costs involved in further drug development.

The MTT assay and other tetrazolium reduction assays have often been used to assess the cytotoxicity of anti-TB drugs, new chemical entities, or combinations (48, 50, 84–86). However, the major drawback of viability assays is that they only focus on the effects of the drugs on one aspect of metabolism, such as the generation of oxidized reducing equivalents contributing to the viability of the cells and are not sensitive enough to detect alterations to the health of the cell that would impact the functioning of the cell in the absence of cell death. Yet, it is not clear what parameters define the health of the eukaryotic cell or how they can be measured. As energy in the form of ATP is required by all eukaryotic cells to survive and function, perturbations of bioenergetic metabolism, specifically OXPHOS and glycolysis, that cannot be compensated for by the cell will affect both the health and functioning of the cell. Here, we demonstrate how extracellular flux analysis that measures OCR, an indirect measurement of OXPHOS, and ECAR, an indirect measurement of glycolysis, gives noninvasive, real-time rapid insight into how anti-TB drugs affect the bioenergetic health of the cell in the absence of cell death. Our data reveal the increased sensitivity of the extracellular flux analysis by the greater degree of variation in the response of the bioenergetic parameters of the anti-TB drug-treated cells in comparison to the percent viability as the readout of the MTT assay (Fig. 6F and G). This strongly suggests that the bioenergetic parameters detect the effect of drugs on energy metabolism at much earlier time points prior to cell death, which is measured by the MTT viability assay. This was supported by correlation analysis in which the MTT assay had low, and in some cases, negative, correlations with the bioenergetic parameters (Fig. 6A to D), together with our PCA that demonstrated the MTT percent viability clustered separately from all the bioenergetic parameters (Fig. 6E). Due to the large numbers of parameters generated by this study, probability testing was not conducted, instead, hierarchical clustering, PCA, and Pearson’s correlation analysis were used to assess contributions and trends in the parameters measured.

Mitochondrial toxicity is now widely accepted as a common mechanism underlying drug-induced organ toxicities (29, 31, 87) and is being increasingly detected in early stages of drug development using extracellular flux analysis (37, 38, 42). Mitochondrial toxicity of compounds is often assessed by growing the cells in the presence of high galactose (30, 42, 58), which forces the cells to use OXPHOS to produce ATP, thereby increasing the cells’ sensitivity to the effects of mitochondrial toxicants compared to that of cells grown in glucose (88, 89). However, the glucose-galactose switch does not sensitize all cell types to mitochondrial cytotoxicity (90). Furthermore, in our study, we used glucose in our media to allow the cells to shift to glycolysis for ATP production (as evidenced by an increase in ECAR) should the drug adversely affect mitochondrial respiration (revealed by decreased OCR). Cells which cannot increase glycolysis in response to drug-impaired mitochondrial respiration will not be able to meet the ATP requirements of the cell, thus increasing the cell’s susceptibility to adverse effects of the drug. Galactose does not allow this switch from OXPHOS to glycolysis. For this reason, we investigated the effects of the anti-TB drugs on hepatocytes, which rely more on OXPHOS, in addition to macrophages, which are more glycolytic, in the presence of glucose. Furthermore, we used the Cell Mito Stress Test in the extracellular flux analysis, as it has been demonstrated that the addition of the mitochondrial stressors oligomycin, FCCP, antimycin A, and rotenone increase the sensitivity of the assay to detect mitochondrial dysfunction (36).

Addition of one of these stresses, FCCP, uncouples respiration from the production of ATP, resulting in maximal respiration (max resp) as a measure of the maximal activity of the electron transport chain (91). This enables the measurement of the SRC, which has been reported to be a marker of cellular stress and mitochondrial dysfunction (92, 93). The SRC clustered separately from the other bioenergetic parameters in the hierarchical clustering of the effects of the anti-TB drugs on the bioenergetic parameters of the THP-1 cells (Fig. 3C), hMDMs (Fig. 3D), and all three cell types combined (Fig. 3A), demonstrating that treatment with the anti-TB drugs perturbs SRC differently than it perturbs the other parameters. This is further supported by the correlation analyses of the different bioenergetic parameters, where the average Pearson’s correlation coefficient was the lowest for SRC (Fig. 4A) in the THP-1 cells and hMDMs and when the parameters of all three cell types were combined. Additionally, SRC also clustered separately from the other bioenergetic parameters in our PCA of the bioenergetic parameters and the MTT percent viability (Fig. 6E), again reinforcing the distinctness of the SRC from the other bioenergetic parameters. Altogether, this demonstrates the high sensitivity of SRC to effects of the anti-TB drugs on the mitochondria. This has been supported by a study investigating the high-throughput respirometry potential of the XF assay to detect mitochondrial biogenesis and toxicity (38). Known mitochondrial toxicants caused concentration-dependent depression in FCCP-uncoupled OCR with no significant decrease in the basal respiration of rabbit renal proximal tubule cells (RPTCs). The authors concluded that the FCCP-uncoupled OCR can be used to uncover disrupted electron transport activity, and consequently mitochondrial damage, by toxicants even though basal metabolism is not impaired (38). As SRC is calculated from the response of OCR after addition of FCCP, our findings suggest that SRC can also be used to identify mitochondrial damage. Other studies investigating drug-induced cytotoxicity have also used FCCP-uncoupled OCR together with changes in initial OCR and ECAR in response to acute treatment or longer incubations with the drug for RPTCs (89) and for RPTCs and HepG2 cells (90) to specifically detect mitochondrial toxicity.

The choice of cell type to investigate cytotoxicity in drug development depends heavily on the environment, whether it be in industry or academia. A limitation of working with primary cells is the variability observed between cells from different donors. To overcome this, we isolated monocytes from the buffy coats of three donors and mixed them prior to seeding them into the microtiter plates. However, this induces a further limitation where the number of replicates (*n*) is then equal to one. Overall, we found the bioenergetic parameters of the macrophage models (THP-1 cells and hMDMs) were much more sensitive to the anti-TB drugs than those of the HepG2 cells (see Data Set S1 in the supplemental material). This may be due to HepG2 cells having a greater SRC than the macrophages, enabling them to tolerate further mitochondrial toxic insults than the macrophages with a lower SRC. This increased sensitivity is underscored by the distinct clustering of the effects of the of the anti-TB drugs on the bioenergetic parameters of the THP-1 cells, followed by broader groupings in hMDM cells and the least distinction in the HepG2 cells (Fig. 5). These differences are very likely due to the differences in metabolism between the cell types, namely, hepatocytes, which are more oxidative, versus the metabolism in macrophages, which are more glycolytic. Specifically, the differences between the two macrophage models, which we have previously observed (71), are expected due to the metabolic differences between primary cells and cell lines; however, similarities in the clustering of the effects of the drugs on their bioenergetic parameters is also notable. These findings suggest that cytotoxic potential of new drug leads should not be investigated on one cell type alone. Although HepG2 cells give an indication of potential hepatotoxicity of early drug leads, macrophages are important in the innate immune response to M. tuberculosis infection and activation of the adaptive immune response. As the metabolism of the immune cells determines the immune functions of the immune cells, any alterations of the bioenergetic metabolism by new TB drug leads indicate the potential of these drugs to attenuate the immune response to M. tuberculosis. Our findings strongly advocate the use of two cell types for the screening of cytotoxicity in TB drug development: HepG2 cells for detection of drug-induced liver toxicity and a macrophage model to detect the effects of the anti-TB drugs on immune cells, which are essential for control of infection.

Strikingly, in all three cell types, the effects of EMB or MXF on the bioenergetic parameters of the cell types either group away from or cluster together with the effects of the other anti-TB drugs on the bioenergetic parameters. These two drugs are not chemically related (see Fig. S3) and do not have similar mechanisms of action on M. tuberculosis (Table 1), which, importantly, suggests that cytotoxic effects cannot be predicted from structure-activity relationships. EMB is thought to inhibit the biosynthesis of the cell wall, by inhibiting arabinosyltransferase required for the synthesis of arabinogalactan and lipoarabinomannan (94), whereas MXF is a fluoroquinolone that inhibits DNA gyrase that allows the untwisting required to synthesize two DNA helices from one DNA double helix (95). This is further supported by the effects of INH and RIF on the bioenergetic parameters clustering together in both the THP-1 and hMDM cells, although they are not chemically related (Fig. S3) and they do not have similar mechanisms of action (Table 1). The clustering of the effects of EMB and MXF in addition to those of INH and RIF on the bioenergetic parameters of the hMDM cells were also mimicked in the hierarchical clustering of the effects of the same drugs on the levels of cytokines produced by hMDMs (Fig. 7). These findings of chemically unrelated drugs inducing similar effects on the bioenergetic parameters as well as similar patterns on hMDM cytokine production caution against associating the cytotoxicity of new chemical entities with their chemical structures or mode of action against M. tuberculosis. Table 1 lists the drugs used in this study and their mechanisms of action.

**TABLE 1:** Mechanism of action of anti-TB drugs examined in this study

Anti-TB drug	Mechanism of action and references
BDQ	Inhibits M. tuberculosis F_1_F_o_ ATP synthase and hence ATP production (106), in addition to modulating respiration (86) and metabolism (107, 108)
CFZ	Competes with menaquinone, a cofactor of the M. tuberculosis electron transport chain, for reduction by NADH dehydrogenase-2 to release reactive oxygen species upon reoxidation to molecular oxygen (109)
RIF	Inhibits M. tuberculosis transcription by binding to M. tuberculosis DNA-dependent RNA polymerase (110)
INH	Inhibits mycolic acid synthesis (111); a prodrug that is activated by KatG (112), and the active form covalently attaches to NAD bound within the active site of InhA, a component of the fatty acid synthase II system of M. tuberculosis, and causes the NADH to dissociate from InhA (113, 114)
PZA	Prodrug that is activated by pyrazinamidase to pyrazinoic acid; the anti-TB activity has been attributed to the disruption of the proton motive force, which is required for essential membrane transport functions, at acidic pH (115)
EMB	Appears to affect the biosynthesis of arabinogalactan by inhibiting polymerization of cell wall arabinan (94).
STR	Binds to the 16S rRNA of the 30S ribosomal subunit, irreversibly affecting polypeptide synthesis and ultimately inhibiting translation (116)
LZD	Binds to the M. tuberculosis 23S rRNA of the ribosomal 50S subunit and prevents the formation of the 70S ribosomal unit and therefore protein synthesis (117)
MXF	Inhibits M. tuberculosis DNA gyrase, preventing DNA replication and transcription (95)

In conclusion, we have adopted real-time extracellular flux analysis to detect early cytotoxic effects of the anti-TB drugs on the health of the cell prior to cell death by assessing the effects of the anti-TB drugs on the bioenergetic parameters of human HepG2 cells, THP-1 macrophages, and hMDMs. In particular, SRC is the most sensitive measure of early mitochondrial toxicity induced by drug treatment. Interestingly, we found that chemically unrelated drugs with differing modes of action on M. tuberculosis cluster together according to their similar effects on the bioenergetic metabolism of the cells, in particular, THP-1 cells. This points to the prudence of associating chemical structure and mode of action on M. tuberculosis with potential cytotoxicity patterns. Furthermore, our findings strongly advocate measuring the effects of new drug leads on the bioenergetic metabolism of macrophages in addition to HepG2 cells to assess cytotoxicity, as this will not only assess hepatotoxicity but will also give an early indication of the potential of the new drug leads to modulate the immune functions of immune cells that might pose a risk to controlling M. tuberculosis infection. Thus, these findings can be used to establish a benchmark for cytotoxicity testing in future TB drug discovery.

## MATERIALS AND METHODS

### Tissue culture and differentiation.

**(i) Human monocyte-derived macrophages.** Peripheral blood mononuclear cells (PBMCs) were isolated from buffy coats (South African National Blood Service). Briefly, 8 ml buffy coat was diluted in 27 ml Dulbecco’s phosphate-buffered saline (DPBS) and overlaid onto 15 ml Histopaque 1077. The buffy coat was separated (400 × *g*, 35 min, swing-out bucket rotor, no acceleration, no brake). The PBMC-enriched layer was collected and washed with DPBS (1:1). PBMCs were pelleted (400 × *g*, 10 min) and washed in 50 ml DPBS (room temperature), and then the wash was repeated with 50 ml DPBS (4°C). PBMCs were pelleted and resuspended in 5 ml separation buffer (DPBS, 2 mM EDTA, 0.5% [wt/vol] bovine serum albumin [BSA], 4°C). CD14^+^ monocytes were isolated by magnetic cell sorting using MACS CD14-microbeads (Miltenyi, 130-505-201) according to the manufacturer’s instructions. The monocytes were pelleted and resuspended in freezing solution (RPMI 1640 medium with final concentrations of 10% [vol/vol] human serum, 1 mM sodium pyruvate, 10 mM HEPES, 1× nonessential amino acids, 2 mM GlutaMAX, and 10% [vol/vol] dimethyl sulfoxide [DMSO]). For each experiment, monocytes isolated from three donors were thawed in cell culture medium (RPMI 1640 medium, 10% [vol/vol] human serum, 1 mM sodium pyruvate, 10 mM HEPES, 1× nonessential amino acids, 2 mM GlutaMAX), mixed, and counted using trypan blue to assess the viability; then, the cells were seeded directly into XFe96 cell microtiter plates at a density of 8 × 10^4^ cells per well in a volume of 80 μl. The monocytes were terminally differentiated into macrophages with 100 ng/ml GM-CSF for 6 days, with a medium change (including the GM-CSF) on day 4. On the sixth day, the macrophages were treated with the anti-TB drugs for 24 h prior to extracellular flux analysis on the XFe96 and viability analysis using the MTT assay. Monocytes from three donors were mixed prior to plating due to the high numbers of cells that were required to seed the XFe96 microtiter plates and to reduce the variability between different donors.

### (ii) THP-1 macrophages.

THP-1 monocytes (ATCC TIB-202) were cultured in RPMI 1640 medium (final concentrations: 10% [vol/vol] fetal bovine serum [FBS], 25 mM d-glucose, 10 mM HEPES, 1 mM sodium pyruvate, 2 mM GlutaMAX, and 0.05 mM β-mercaptoethanol) under standard tissue culture conditions (37°C, 5% CO_2_). Cells were washed in fresh medium, counted, seeded in the XFe96 cell culture plate at a density of 100,000 cells per well in 80 μl RPMI 1640 culture medium, and terminally differentiated with 25 nM phorbol 12-myristate-13-acetate (PMA) for 3 days. On the fourth day, fresh medium without PMA was supplied to the cells, and on the fifth day, the cells were treated with the anti-TB drugs for 24 h.

### (iii) HepG2 cells.

HepG2 cells (ATCC HB-8065) were cultured in Dulbecco’s modified Eagle medium (DMEM) supplemented with 10% (vol/vol) FBS. To seed, cells were washed with warm DPBS and lifted with warm 1× trypsin-DPBS. Trypsin was deactivated with the addition of culture medium. Cells were harvested, pelleted (400 × *g*, 5 min), resuspended in fresh medium, and seeded at a density of 25,000 cells per well in 80 μl DMEM culture medium in the XFe96 cell microtiter plate. Cells adhered naturally overnight, followed by 24 h treatment with the anti-TB drugs.

### Anti-TB drug treatment and Agilent Seahorse Cell Mito Stress Test.

Stock solutions of anti-TB drugs were prepared in DMSO or DPBS where possible (Table 2). Working drug solutions were prepared in the respective media, and the final concentration of DMSO per well did not exceed 0.2% (vol/vol), except for the highest concentrations (50× and 100× MICs) of clofazimine and linezolid (0.5% and 1% [vol/vol]), in which case 0.5% and 1% DMSO controls were included in the assays. Following seeding and/or differentiation of each cell type, the supernatant was aspirated, and the cells were treated with four concentrations of each anti-TB drug: 1×, 10×, 50×, and 100× the MIC values in Table 2 in 8 replicates for 24 h in a total volume of 80 μl/well. Cells were also treated with two drug combinations: (i) INH, RIF, PZA, EMB, and STR or (ii) INH and RIF at 1× and 10× MICs of all the drugs in the combination for 24 h. The following day, the cells were washed twice with CMST medium (DMEM, 30 mM NaCl, 5 mM HEPES, 2 mM GlutaMAX, and 1 mM sodium pyruvate, pH 7.4), and the final volume was brought up to 180 μl with CMST medium. The medium in the cell plate was degassed for a minimum of 30 min in a non-CO_2_ incubator. The mitochondrial modulators oligomycin, carbonyl cyanide-4 (trifluoromethoxy) phenylhydrazone (FCCP), rotenone, and antimycin A were prepared in CMST medium from DMSO stocks at 10× the concentrations given in Table 3. The pH of the solutions was adjusted to 7.4 at 37°C and loaded into the ports of the XFe96 cartridge as indicated in Table 3. The extracellular flux of the cells was analyzed on an XFe96 using the Cell Mito stress test (CMST) protocol with 3 min of mixing and 4-min measurements (71, 96).

**TABLE 2:** Concentrations and preparation of anti-TB drugs

Anti-TB drug	Mol wt (g/mol)	Concn (1× MIC)		Reported *in vitro* MIC (reference)	Solvent
		Molarity	Mass/vol		
Bedaquiline fumarate (BDQ)	671.5	44.5 nM	29.88 ng/ml	0.06 μg/ml^*a*^ (97)	DMSO
Clofazimine (CFZ)	473.4	0.2 μM	94.63 ng/ml	0.1 μg/ml (98)	DMSO
Rifampicin (RIF)	822.94	0.4845 μM	398.75 ng/ml	0.1–0.4 μg/ml (99)	30% (vol/vol) DMSO-DPBS
Isoniazid (INH)	137.14	0.23975 μM	32.88 ng/ml	25 ng/ml (100)	DPBS
Pyrazine carboxylamide (PZA)	123.11	0.406 mM	50 μg/ml	6–200 μg/ml (pH dependent) (101)	DPBS
Ethambutol-HCl (EMB)	277.23	2.45 nM	679.2 ng/ml	0.5 μg/ml^*b*^ (102)	DPBS
Moxifloxacin-HCl (MXF)	437.89	1.14 μM	500 ng/ml	0.5 μg/ml (103)	DPBS
Streptomycin sulfate salt (STR)	728.69	172 nM	125 ng/ml	1 μg/ml^*c*^ (104)	DPBS
Linezolid (LZD)	337.35	2.96 μM	1 μg/ml	0.25 μg/ml (105)	30% (vol/vol) DMSO-DPBS

a MIC for BDQ (M = 555.51 g/mol).

b MIC for EMB (M = 204.31 g/mol).

c MIC for streptomycin A (M = 581.57 g/mol).

**TABLE 3:** Final concentrations of mitochondrial modulators used for CMST

Modulator	Port^*a*^	Vol (μl)^*b*^	Concn (μM) used for:		
			hMDM	THP-1	HepG2
Oligomycin	A	20	1.5	1.5	3
FCCP	B	22.5	1	1	2.5
Rotenone and antimycin A	C	25	2.5	0.5	0.5

a Port on XFe96 cartridge.

b Volume loaded into the port.

### Normalization of extracellular flux by protein concentration.

Following XFe96 analysis, the supernatants were aspirated from all the wells, leaving behind approximately 10 μl of the supernatant in each well, and the cells were fixed with the addition of 10 μl formalin/well. The cells in each well were lysed by adding 20 μl 25 mM NaOH/well. BSA standards (5 μl) were added to the control wells without cells (lanes 1 and 12) ranging from 0.125 to 2 mg/ml (Bio-Rad 500-0202) and treated with formalin and NaOH at the same concentrations as used for the cells. Bradford reagent (150 μl, Bio-Rad 500-0205) was added to all the wells, and the plate was incubated in the dark for 5 min. The absorbance of each well was measured at 595 nm using a Biotek Synergy H4 Hybrid spectrophotometer, and the standard curve was generated from the BSA standards in lanes 1 and 12 was used to calculate the protein concentrations in each well. These protein concentrations were used to normalize the bioenergetic parameter data (OCR and ECAR) with the Agilent Seahorse Wave desktop software (version 2.6). The CMST assay parameters were calculated using the CMST Report Generator and exported using the Agilent Seahorse Biosciences Cell Mito stress test report generator for further statistical analysis.

### MTT viability assay.

To assess the viability of the cells under conditions identical to those used for the extracellular flux analysis, the cells were seeded into XFe96 cell microtiter plates and treated as for the XFe96 assay. Media in lanes 1 and 12 of the microtiter plate served as the negative controls. The MTT reagent (3-[4,5-dimethylthiazol-2-yl]-2,5-diphenyltetrazolium bromide; Invitrogen M6494) was prepared and the assay was performed according to manufacturer’s instructions. Briefly, after overnight drug treatments, the supernatant was aspirated, leaving 25 μl of supernatant behind to avoid lifting the cells. The volume was brought up to 100 μl with the appropriate medium, and 10 μl of MTT reagent (5 mg/ml in DPBS) was added to each well and incubated for 4 h (37°C, 5% CO_2_). Supernatant was aspirated, and the formazan crystals was dissolved with 50 μl DMSO in each well followed by a 10-min incubation at room temperature. The absorbance of each well at 540 nm was measured using a Biotek Synergy after mixing by trituration. The percentage viability of the cells was calculated as follows:

$$\text{\% viability}=\frac{\text{mean OD of drug}-\text{treated cells}-\text{mean OD of negative control}}{\text{mean OD of untreated cells}-\text{mean of negative control}}\times 100,$$

where OD is the optical density.

### Cytokine measurements in the culture supernatant fluid.

Culture supernatant was collected from the hMDM cells treated with the anti-TB drugs for 24 h prior to XF runs and stored at −80°C. The cytokine levels were measured using a magnetic bead-based Bio-Plex Pro Human cytokine 27-Plex (Bio-Rad) using the Bio-Plex 200 instrument according to the manufacturer’s instructions. Using the Bio-Plex Manager Software and standard curves of each cytokine, the concentrations of the cytokines (pg/ml) were calculated from the median fluorescence intensity (MFI). Four replicates were used for each concentration of each anti-TB drug analyzed.

### Statistical analyses.

Two-way analyses of variance (ANOVAs) were performed using GraphPad Prism for the bioenergetic parameters. As numerous parameters were generated from the bioenergetic and viability analyses of three cell types treated with nine drugs at four different concentrations, probability testing was not conducted; instead, principal-component analysis and hierarchical clustering were undertaken to discern contributions and trends in the measured parameters. Principle-component analysis (PCA), hierarchical clustering (heat map), and Pearson’s correlation were performed using software package Partek Genomic Suite (PGS; Partek, St. Louis, MO) according to factory settings and the user manual. *P* values of less than 0.05 were considered significant. Briefly, z normalization was performed before hierarchical clustering. Euclidean dissimilar matrix and average linkage similarity were used. In PCA, all variables were assumed to have equal influence on principle components (PCs).
