# Supplementary material for: Host Bioenergetic Parameters Reveal Cytotoxicity of Antituberculosis Drugs Undetected Using Conventional Viability Assays
Source: Antimicrob Agents Chemother. 2021 Sep 17;65(10):e00932-21. doi: 10.1128/AAC.00932-21 (PMC8448146; doi:10.1128/AAC.00932-21)
Supplement: Supplemental file 1 — Fig. S1 to S3. Download AAC.00932-21-s0001.pdf, PDF file, 0.5 MB [file aac.00932-21-s0001.pdf]

Supplementary Information

Host bioenergetic parameters reveal cytotoxicity of anti-TB drugs undetected using conventional viability assays.

Bridgette M. Cumming[1], Zainab Baig[1], Kelvin W. Addicott[1], Dongquan Chen[2], Adrie J.C. Steyn[1,3,4]

[1]Africa Health Research Institute, University of KwaZulu-Natal, Durban, KwaZulu-Natal, South Africa
[2]Division of Preventive Medicine and Comprehensive Cancer Center. University of Alabama at Birmingham, Birmingham, AL, USA
[3]Department of Microbiology, University of Alabama at Birmingham, AL, USA
[4]Centers for AIDS Research and for Free Radical Biology, University of Alabama at Birmingham, Birmingham, AL, USA

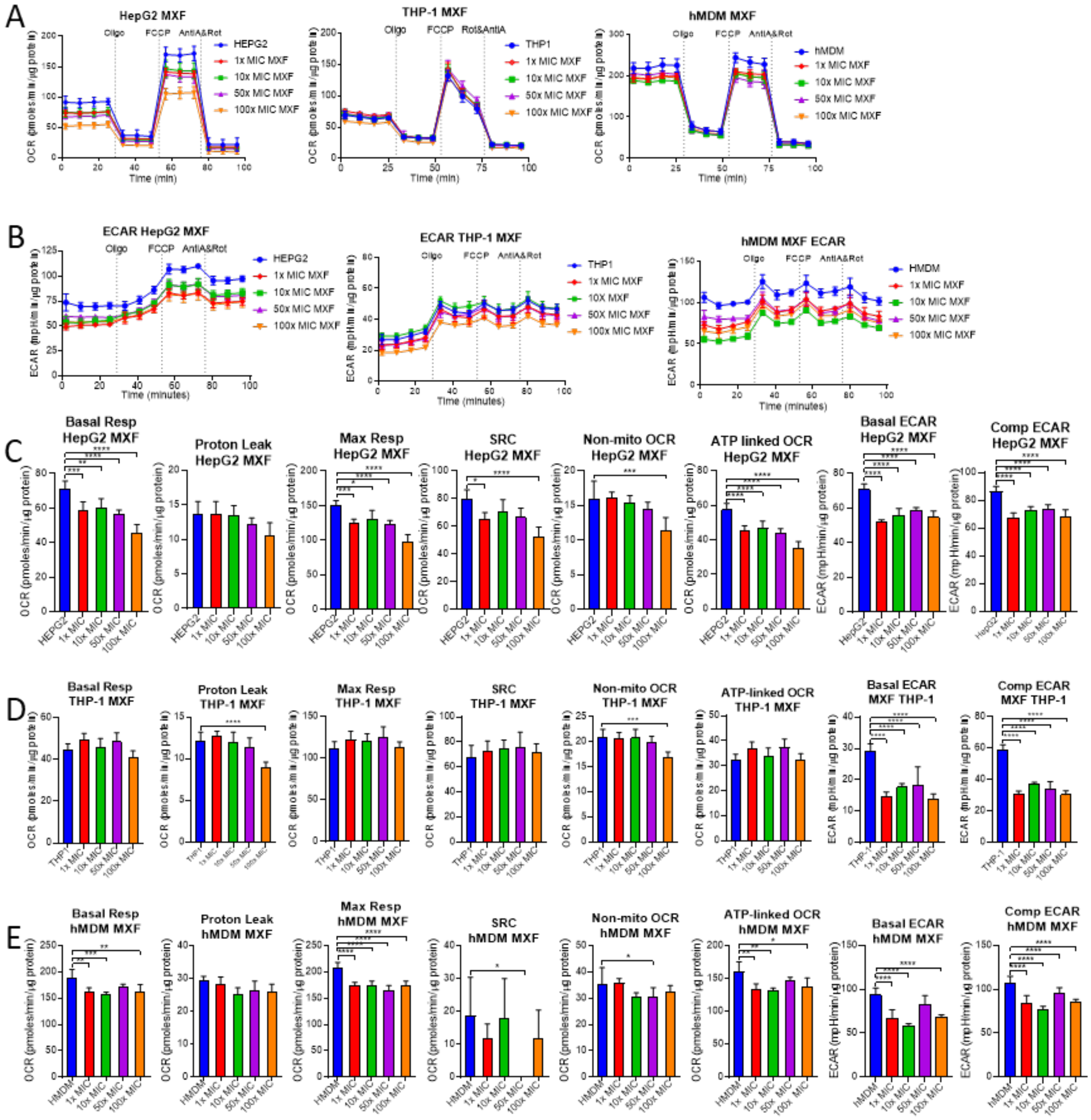

Figure S1: CMST and ECAR profiles and bioenergetic parameters of cells treated with MXF at increasing MIC. (A) CMST and (B) ECAR profiles of HepG2, THP-1 and hMDM cells, respectively, treated with increasing MIC of MXF. The profiles were used to calculate the indicated bioenergetic parameters of the (C) HepG2, (D) THP-1, and (E) hMDM cells, respectively. Monocytes isolated from the buffy coats of three donors were mixed and used for each experiment (n =1).

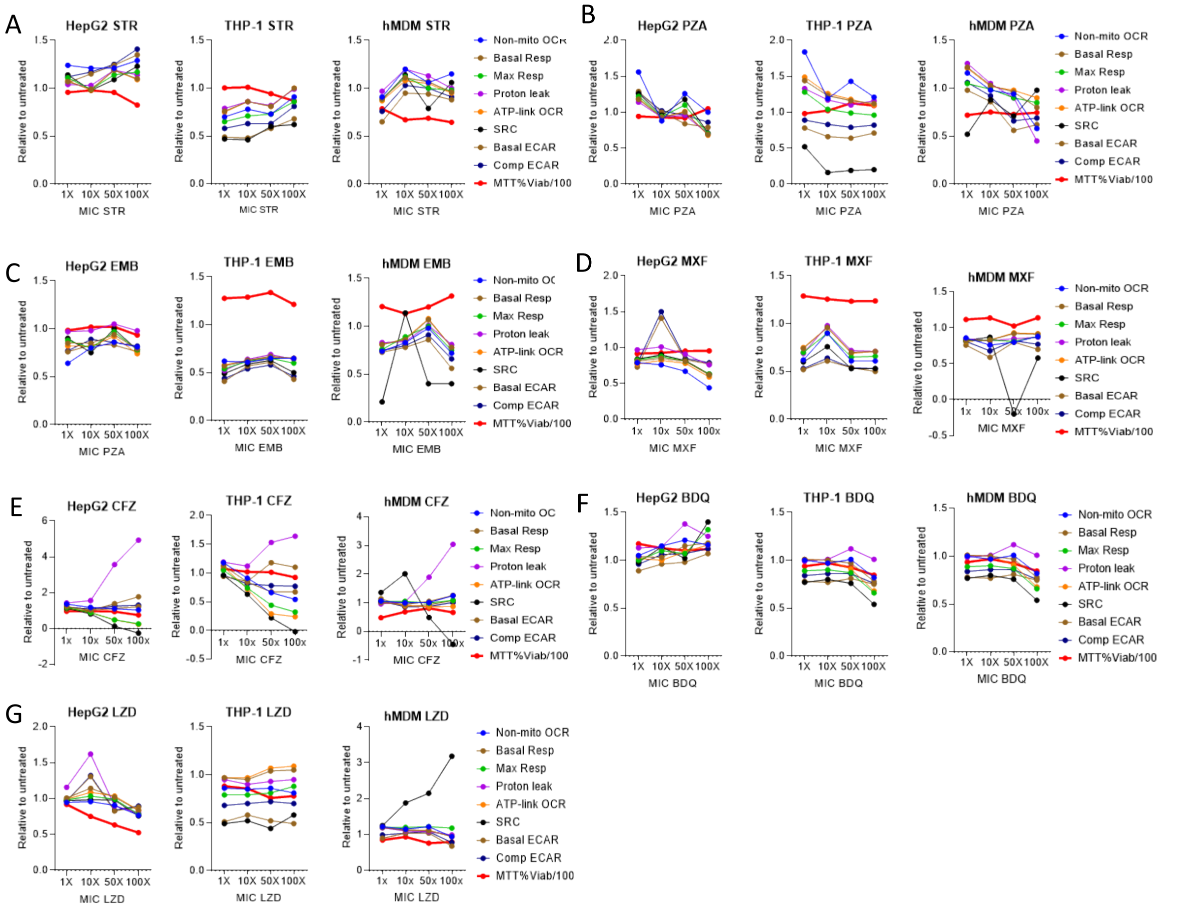

Figure S2: Comparison of line plots of the bioenergetic parameters of anti-TB drug-treated cells relative to untreated cells with that of the MTT %Viability of the same cells. The line plots demonstrate that the relative bioenergetic parameters of HepG2, THP-1 and hMDM cells treated with increasing MIC of (A) STR, (B) PZA, (C) EMB, (D) MXF, (E) CFZ, (F) BDQ and (G) LZD indicate a broader range of effects of the drugs than %Viability alone.

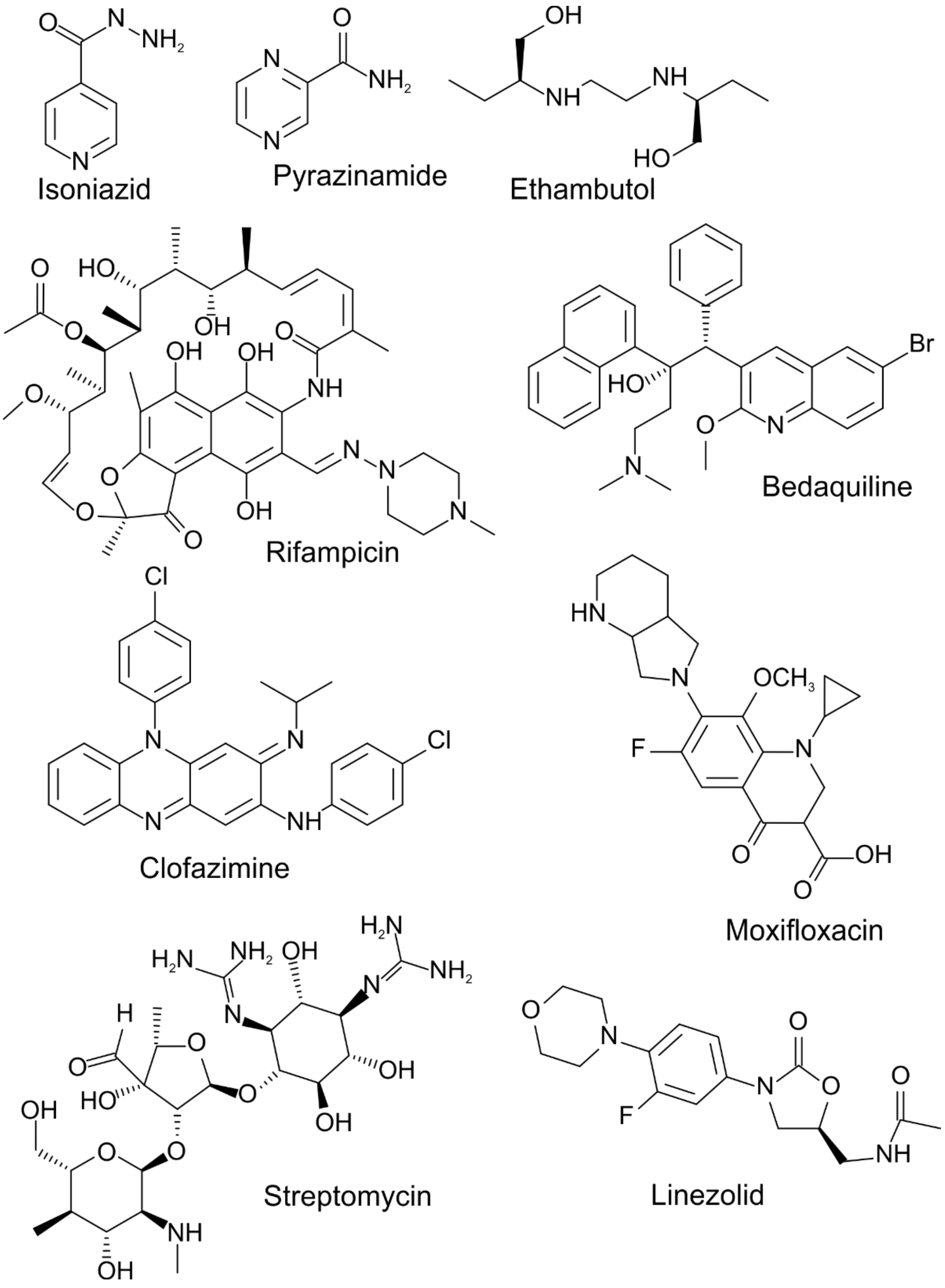

Figure S3. Structures of anti-TB drugs
